# Mechanical and microstructural performance of concrete containing high-volume of bagasse ash and silica fume

**DOI:** 10.1038/s41598-022-08749-1

**Published:** 2022-04-06

**Authors:** Muhammad Nasir Amin, Afaq Ahmad, Khan Shahzada, Kaffayatullah Khan, Fazal E. Jalal, Muhammad Ghulam Qadir

**Affiliations:** 1 Department of Civil and Environmental Engineering, College of Engineering, King Faisal University, Al-Ahsa, 31982 Saudi Arabia; 2 Department of Civil Engineering, University of Engineering and Technology, Peshawar, Pakistan; 3 Department of Civil Engineering, Shanghai Jiao Tong University, Shanghai, 200240 People’s Republic of China; 4 Department of Environmental Sciences, COMSATS University Islamabad, Abbottabad Campus, Abbottabad, 22060 Pakistan

**Keywords:** Engineering, Materials science

## Abstract

In this study, researchers examined the effect of replacing a high-volume of cement with sugarcane bagasse ash (BA) and silica fume (SF). In addition to the control, three binary and three ternary blends of concrete containing different percentages of cement/BA and cement/BA/SF were tested to determine the various mechanical and microstructural properties of concrete. For each mix, eighteen cylindrical concrete specimens were cast followed by standard curing (moist at 20 °C) to test the compressive and tensile strengths of three identical specimens at 7, 28, and 91 days. The test results indicated that the binary mix with 20% BA and ternary mix with 33% BA and 7% SF exhibited higher strengths than all the other mixes, including the control. The higher strengths of these mixes are also validated by their lower water absorption and apparent porosity than the other mixes. Following mechanical testing, the micro and pore structures of all mixes were investigated by performing scanning electron microscopy/energy-dispersive X-ray spectroscopy (SEM–EDS), Fourier transform infrared (FTIR) spectroscopy, thermogravimetric analysis (TGA), and nitrogen (N_2_) adsorption isotherm analysis. In SEM–EDS analysis, a dense and compact microstructure was observed for the BA20 and BA33SF7 mixtures due to the formation of high-density C–S–H and C–H phases. The formation of a large amount of C–S–H phases was observed through FTIR, where a prominent shift in peaks from 955 to 970 cm^−1^ was observed in the spectra of these mixes. Moreover, in N_2_ adsorption isotherm analysis, a decrease in the intruded pore volume and an increase in the BET surface area of the paste matrix indicate the densification of the pore structure of these mixes. As observed through TGA, a reduction in the amount of the portlandite phase in these mixes leads to the formation of their more densified micro and pore structures. The current findings indicate that BA (20%) and its blend with SF (40%) represents a potential revenue stream for the development of sustainable and high-performance concretes in the future.

## Introduction

Concrete, a synthetic rock comprising Portland cement, fine aggregate, coarse aggregate and water, is the basic building block of the urbanizing world. It is a widely used human-made commodity in the construction industry, with global annual production exceeding 25 billion metric tons^1,2^. The widespread use of concrete is associated with its proven flexibility, adaptability, prevalent availability, and impermeable nature. Over the past six decades, the consumption of cement has increased tenfold^3^. According to Statista, over 4.5 billion tons of Portland cement was utilized in the development of infrastructure in 2019, thereby producing 3.5 billion tons of CO_2_^4^. Additionally, the production and consumption of cement has reduced natural resources and has also led to increased consumption of energy, which has a negative effect on the environment^1^. According to the 2017 Global Status Report released by the United Nations, CO_2_ emissions in the construction industry increased by approximately 1% per year between 2010 and 2016, thereby liberating 76 GtCO_2_ in cumulative emissions such that building-related CO_2_ release rose by almost 1% annually during the last decade, leading to four million deaths annually^5^. The cement industry is presently the third-largest sector to consume energy and the second-largest sector to globally emit CO_2_^6^, with the production of 1 ton of ordinary Portland cement (OPC) emitting almost 1 ton of CO_2_^7^. Multiple techniques have been suggested to decrease the high energy demand and carbon footprint, including carbon capture, clinker reduction, an alternative fuel source, and cement kiln optimization. Comparatively, substitution of clinker with supplementary cementitious materials (SCMs) is the most effective and practical technique^8^. Hence, numerous researchers are exploring appropriate alternatives to partially or fully substitute cement. SCMs are used as partial replacements of cement owing to their pozzolanic characteristics^9^. These materials possess activatable latent hydraulic reactivity and are generally found in agricultural and industrial wastes^1^, demonstrating higher resistance to corrosion^10^. Various agricultural wastes sugarcane bagasse ash (SCBA)^11^, wheat straw ash^12^, rice husk ash (RHA)^13^, palm oil fuel ash (POFA)^14^, etc.) and industrial waste fly ash (FA)^15^, marble dust^16^, electric arc furnace slag (EAFS)^17^, silica fume (SF)^18^, etc.) have been reused as substitutes for cement to attain green concrete with ameliorated properties (higher strength, enhanced workability, cost effectiveness, less heat of hydration, and minimum carbon footprint in the cement industry)^1^. The most commonly used SCMs are FA and GGBFS. However, due to depleting production and quality, their use is decreasing, and as a result, other natural materials that are abundant and sustainable, such as agro-waste ash and volcanic ash, are being researched. To (i) not exceed global warming of 2 °C and (ii) to achieve 100% net-zero carbon buildings by 2050, the emission factor must remain well below 20 tons CO_2_ per TJ by addressing the energy-carbon intensities of present buildings^6^. In lieu of that, replacement of 50% cement in the construction industry before 2030 is also a target of UNDP. Therefore, since the inclusion of more than 20% SCBA leads to a reduction in other mechanical properties, incorporating SF is suggested to regain the lost strength.

Waste generation rates are increasing worldwide because of rapid population growth and urbanization. The annual global production of solid waste is approaching 17 billion tons per year, while it is predicted to reach almost 27 billion tons by 2050, an alarming finding. Mainly, the rapidly growing population of Asia and Africa accounts for this large increase^19,20^. To mitigate environmental nuisance, valorization of these solid wastes to high-value products is extremely imperative^21^. Agro-wastes refer to the residues produced due to the cultivation and processing of crude agricultural products, thus creating non-agro-wastes or industrial wastes. However, agro-wastes bear good physico-mechanical characteristics, are environmentally sustainable, cost-effective, and energy efficient and can therefore be employed in various construction applications^22^. In Europe, the NoAW (No Agro-Waste) project follows the policy of a ‘near zero-waste’ society and is searching for other innovative approaches, thereby allowing the transformation of growing agro-industrial waste (exceeding 250 million tons/year) into eco-efficient bio-based products^23^. To enhance the mechanical properties of concrete, some of the prominent SCMs used are SCBA, RHA, corn cob ash (CCA), POFA, and SF, among others. Worldwide, almost 1500 million tons of sugarcane is produced every year, which leads to almost 40–45% bagasse after juice has been extracted^24^. The annual global production of sugarcane bagasse (a significant renewable energy source) is highest in Brazil^25^ (36%), India^26^ (17%), Thailand (8%), and China (7%), followed by Pakistan (4%)^27^. Sales and Lima^28^ reported that one ton of bagasse can produce almost 25–40 kg of BA, wherein mesoporous silica, the most predominant oxide (∼ 70%), acts as a catalyst and is largely used in the production of geopolymers^29–31^, alongside other commercial applications. Additionally, 0.26 million tons of BA is also being produced annually during industrial fueling processes^32^, thus confirming that SCBA is abundantly available in Pakistan. The SiO_2_ content in SCBA is affected by the amount of silicon available in the soil, whereas sugarcane roots mainly govern the absorption of silicic acid from clay and subsequently transmit it to the shoots in the form of amorphous silica. Nevertheless, most SCBAs are disposed of in landfills^33^, and the use of BA is still propelling currently because of the greater demand for sugar and ethanol worldwide^28^.

ASTM C618 categorizes SCBA as a pozzolanic material because of its higher silica content and crystalline structure^34,35^. According to Zareei et al.^36^ and Neto et al.^37^, the strength and impact resistance of self-compacting concrete (SCC) improved when the alkali-silica reaction of reactive aggregates was mitigated after replacing OPC with 5% BA. In another study, the compressive strength of concrete increased by replacing up to 10% cement with SCBA^30^. The maximum compressive strength (> 160 MPa)^30^, minimal reduction in the formation of Ca(OH)_2_^37^, and superior durability (generating ultrahigh-strength concrete)^38^ were reported by replacing 15 to 20% cement with SCBA, while the bond strength of concrete increased when the SCBA content was between 5 and 30%^26^. In another study, replacement of 5% cement with SCBA increased the average compressive strength by 12%^39^. The higher strength is attributed to the yielding microstructure, which resembles samples with crushed quartz^40^. Several studies have revealed that the flexural strength of concrete and chloride diffusion coefficients decreased while the workability improved with the addition of SCBA^26,34,37,38,41^. Batool et al.^34^ argue that there is utmost need to investigate the salient characteristics and influence of locally available SCBA, whose content generally ranges between 5 and 30%, to produce durable and green concrete/ecological concrete^10,42^. However, Ganesan et al.^43^ and Le et al.^44^ revealed that the slump values decreased at higher SCBA contents. The impact of bagasse ash replacement on concrete workability was also investigated by Venkatesh et al.^45^ and Mahmud et al.^46^. Their results show that slump values for all levels of SCBA replacement concrete were higher than control concrete mix values. In addition to the isolated effect of SCBA, a variety of additives, such as GGBFS, nanosilica and SF, have been added in conjunction with SCBA to attain better performance. While studying the adverse impact of dust on the concrete properties in asphalt pavement, the compressive strength improvement was in the order SF mixes > SCBA mixes > FA mixes^47^. The addition of 10% SCBA enhanced the tensile strength by approximately 10%, thus saving costs and improving durability. In another study, the compressive strength of SCC increased significantly and showed resistance to sulfate attack with 30% SCBA + 30% GGBFS substituting OPC^44^. The inclusion of SCBA (10–30%) in mixtures blended with nanosilica (3–6%) decreased the compressive strength^41^. For a 10% SCBA + 10% micro silica mix, maximum compressive and flexural strengths and improved resistance to acid attack were achieved after curing^48^. Moreover, 15% SCBA + 10% SF was recommended by Teja et al.^49^ to attain an increase in strength (up to 24%).

Larissa et al.^50^ used SCBA and metakaolin (MK) to produce SCC for exposure at temperatures of 200 to 800 °C and revealed that the extent of concrete deterioration at high temperatures was significantly reduced. Additionally, SCBA and MK at contents up to 40% were less susceptible to elevated temperatures, thus producing fewer cracks and reducing strength losses compared to those at room temperature. By incorporating the SCBA-SF content (10 to 30%), it was concluded that the slump, temperature, and volumetric mass decreased with increasing percentages. Additionally, the compressive strengths at 7, 14 and 28 days decreased by 7.6%, 7.2% and 7.1%, respectively. Moreover, the mixture prepared using a 20% SCBA-SF mix had excellent corrosion performance in contrast to that prepared using only OPC^51^. Khan et al.^52^ found that by replacing cement by weight with 10% SCBA and 10% micro silica, the blended concrete exhibited better durability and offered optimal resistance to sulphate and chloride attack. The literature suggests that there exists a lack of research focusing on the usage of SCBA at higher percentages (such as 30 to 40%) in combination with highly reactive materials such as SF without compromising the mechanical properties of the resulting mixture. A comprehensive investigation of the aforementioned performance of SCBA-SF in concrete is scarcely available in the existing literature. Therefore, further development is needed to evaluate the characteristics of fresh and hardened concrete modified to generate efficacious SCM blends to be substituted with highly polluting OPC.

Therefore, the impact of BA and its blend with SF, as a partial replacement of cement, on the mechanical, durability and microstructural characteristics of concrete must be examined. In addition, the physical and chemical characteristics of cement, BA, and SF were determined with X-ray fluorescence (XRF) and X-ray diffraction (XRD) methods. Concrete specimens, namely, control specimens (100% cement), three binary mixes with only BA (10, 20, and 30%) and three ternary mixes with BA along with SF (25% BA + 5% SF, 33% BA + 7% SF and 40% BA + 10% SF), as a partial substitute of cement, were selected. In ternary mixes, a large amount of cement was substituted (up to 50%). Mechanical properties, such as the compressive and split tensile strengths with aging (7, 28 and 91 days), and water absorption (WA) and apparent porosity (AP) after aging for 91 days were evaluated for hardened concrete samples. Eventually, to study the formation of hydration products due to BA and SF replacement in the cement matrix, the isolated effect of BA and blended effect achieved by incorporating SF along with BA on the micro and pore structures of the cement mortar samples were explored by SEM–EDS, FTIR spectroscopy, TGA, and the N_2_ adsorption isotherm method.

## Materials and methods

### Materials

In this study, conventional OPC, complying with ASTM C150, commercially available SF and sugarcane BA processed in the laboratory were used as the main binders for the preparation of concrete specimens. The chemical and physical properties of these binder materials are given in Table 1.

**Table 1:** Physical and chemical properties of cement, BA and SF.

	C	BA	SF
**Physical properties**			
Specific gravity (g/cm^3^)	3.20	2.03	2.23
Blain fineness (m^2^/g)	0.344	–	21.5
**Chemical properties (oxides, % by weight)**			
SiO_2_	21.3	59.3	93.3
Al_2_O_3_	5.56	15.9	–
Fe_2_O_3_	3.24	5.01	0.58
SiO_2_ + Al_2_O_3_ + Fe_2_O_3_^a^	–	80.2	–
CaO	63.4	7.59	1.82
MgO	0.93	1.10	0.28
SO_3_	2.25	0.84	–
K_2_O	0.62	6.60	0.88
Na_2_O	0.13	0.89	0.19
LOI^b^	2.41	2.78	2.25
**Compounds (%)**			
C_2_S	47.9	–	–
C_3_S	24.7	–	–
C_3_A	8.76	–	–
C_4_AF	9.86	–	–

^a^ASTM C618; ^b^LOI = loss on ignition.

**Figure 1:**
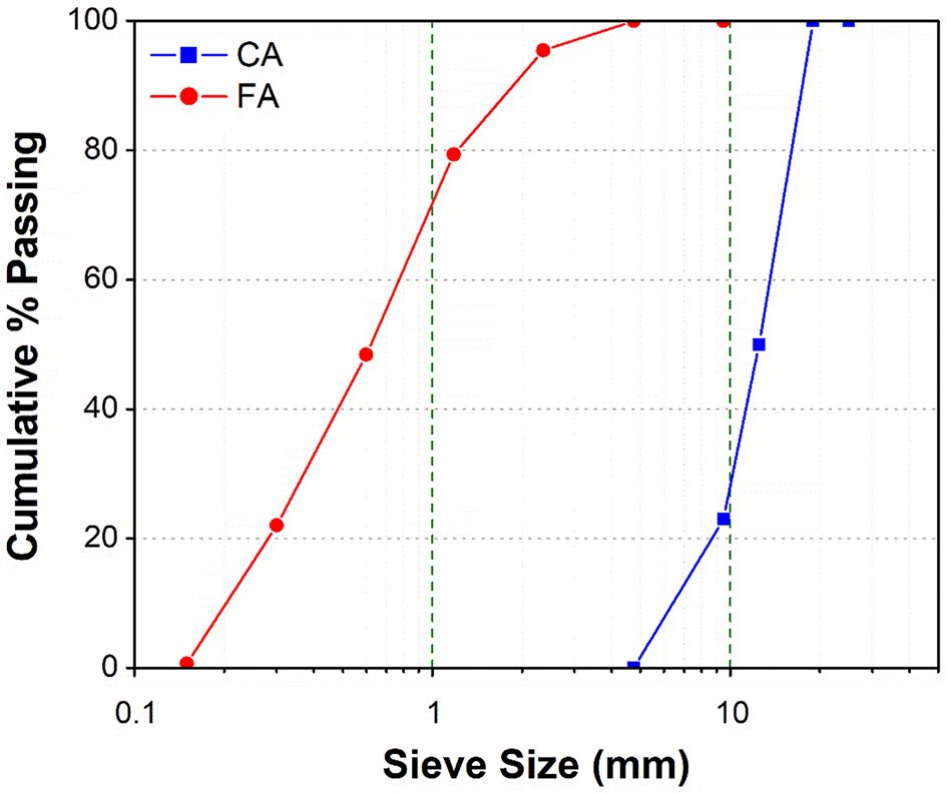
Grain size distribution of coarse and fine aggregates.

In addition to binder materials, locally available coarse and fine aggregates were used as filler materials in concrete. The particle size analysis curves of both fine and coarse aggregates are shown in Fig. 1. The coarse aggregates blending percentages are 50% (20 mm down) and 50% (10 mm down). According to ASTM C33, the specific gravity and water absorption of fine aggregates were 2.60 and 1.03%, respectively, whereas the fineness modulus was calculated to be 2.54 (Table 2). The specific gravity and water absorption of coarse aggregates were 2.65 and 0.82%, respectively.

**Table 2:** Sieve analysis results of coarse and fine aggregates (ASTM C136).

Sieve #	Sieve size (mm)	Weight retained (g)	Weight retained (%)	Cumulative passing (%)	Cumulative retained (%)
**Coarse aggregate (CA)**					
1 inch	25	0	0	100	0
3/4 inch	19	0	0	100	0
1/2 inch	12.5	1552	50.5	50	50
3/8 inch	9.5	803	26.1	23	77
No. 4	4.75	719	23.4	0	100
**Fine aggregate (FA)**					
3/8 inch	9.5	0	0	100	0
No. 4	4.75	0	0	100	0
No. 8	2.36	27	4.54	95.46	4.54
No. 16	1.18	96	16.1	79.33	20.67
No. 30	0.600	184	30.9	48.40	51.60
No. 50	0.300	157	26.4	22.02	77.98
No. 100	0.150	127	21.3	0.67	99.33
Pan	–	4	0.67	–	–
Fineness Modulus of FA **(FM**) = (0 + 4.54 + 20.67 + 51.6 + 77.98 + 99.33)/100 = **2.54**					

#### Burning and grinding of bagasse ash

**Figure 2:**
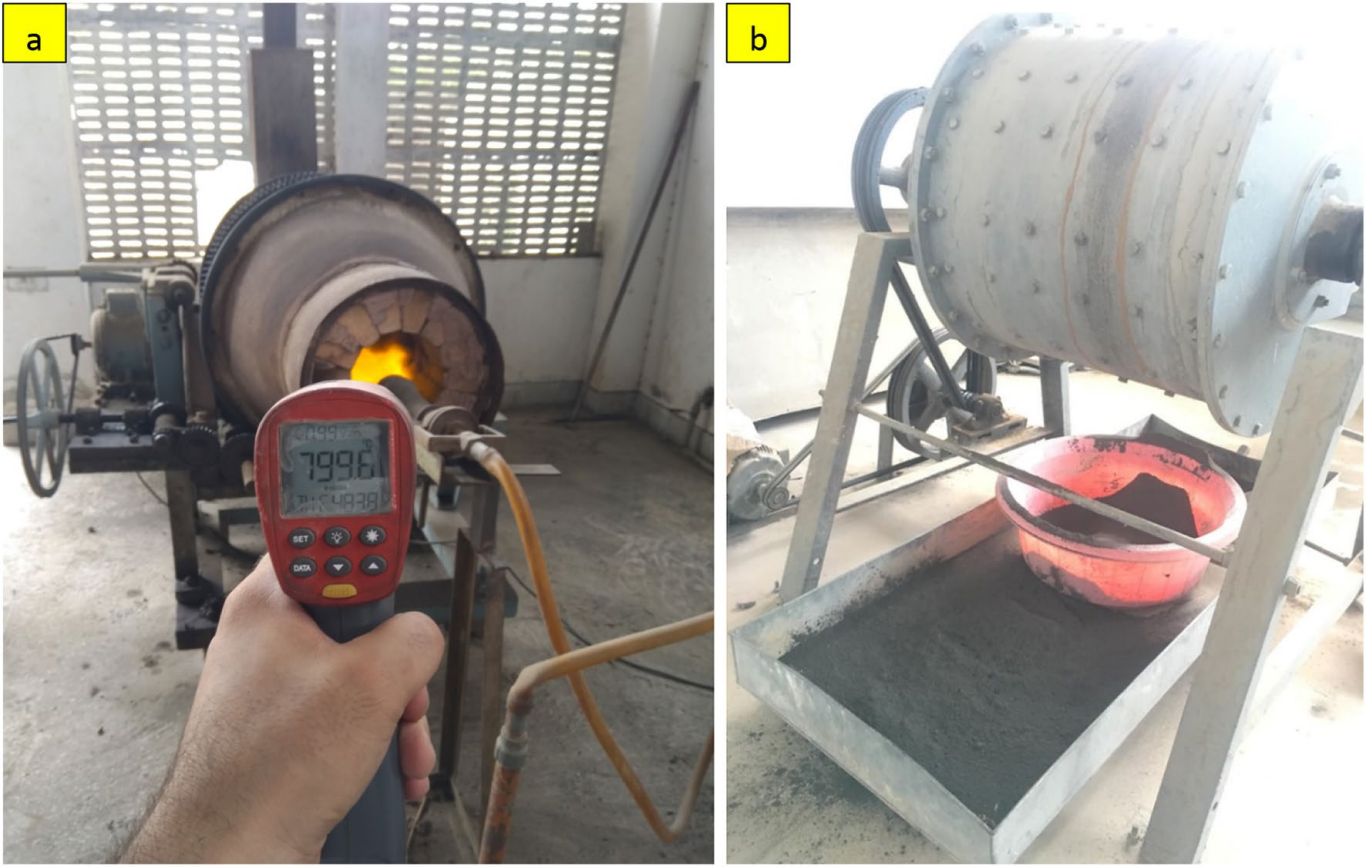
(**a**) Rotary kiln used in burning BA at different temperatures (600 °C and 800 °C), and (**b**) Rotary ball mill used in grinding of BA.

**Figure 3:**
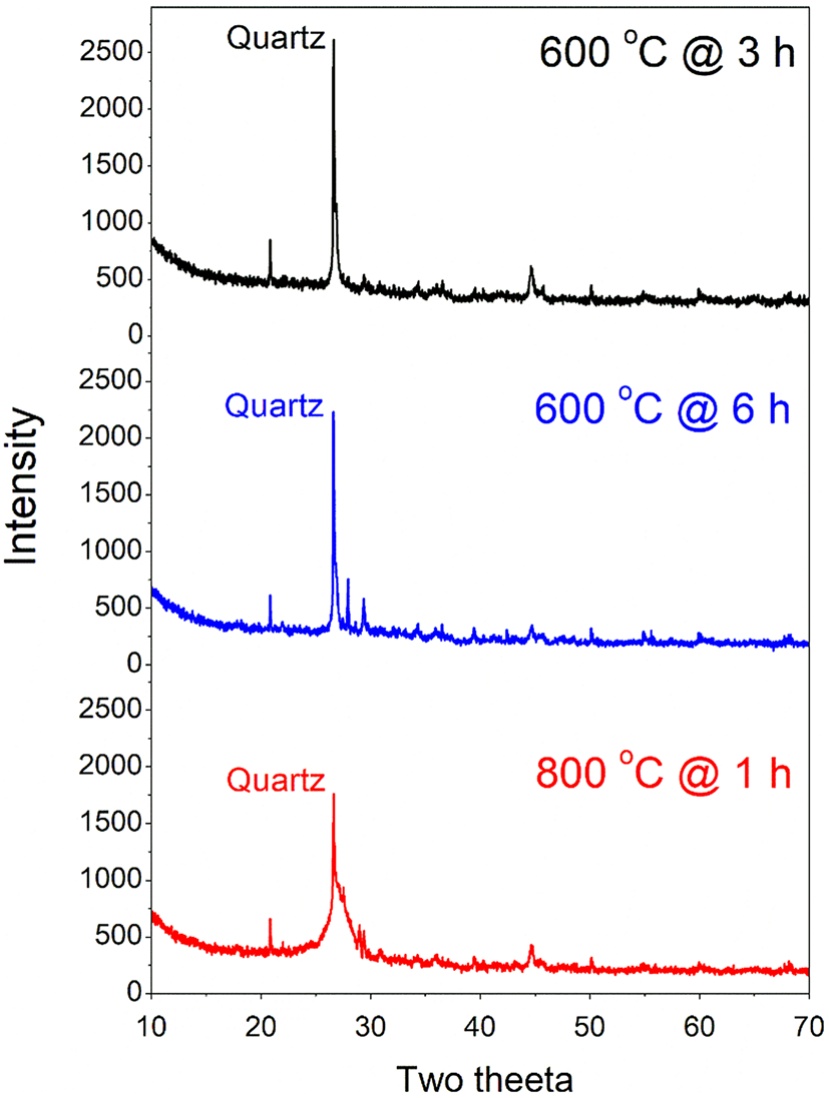
XRD pattern of BA after treating at temperature of 600 °C (@ 3 h and 6 h) and 800 °C (@ 1 h).

The chemical properties of BA mainly depend on its source, organic composition, and sintering temperature. The chemical composition of bagasse husks also varies from region to region and depends on climatic conditions^53^. The obtained sugarcane bagasse husk was burned in an electric furnace at different controlled temperatures. To achieve silica-rich BA, several combinations of burning duration and sintering temperature were employed. This is because the incineration time and temperature significantly affect the quality of BA^54–56^. Therefore, in this study, sugarcane bagasse husks were burned in a heating kiln (Fig. 2a) at different controlled temperatures of 600 °C for 3 h and 6 h and a higher temperature of 800 °C for an hour. Subsequently, XRF and XRD analyses were performed on the powdered samples to study the effect of different burning temperatures on the chemical and mineralogical compositions of BA. The chemical composition of all three BA samples shows that the sum of the SiO_2_, Al_2_O_3_, and Fe_2_O_3_ contents is a maximum (70%), corresponding to a burning temperature of 800 °C. The XRD analysis shown in Fig. 3 demonstrates the amorphous nature of BA due to the silica present in the sample. Based on the chemical composition and XRD results, the BA obtained from burning at 800 °C was selected for grinding to obtain a fine powder. Thus, after cooling under normal air, the BA was subjected to grinding in a rotary ball mill at 15 rpm for 12 h (Fig. 2b). This ground BA was then used as a partial substitute for cement to prepare binary (only BA) and ternary concrete specimens (BA and SF).

#### Concrete mixture proportions

Including the control, a total of seven different concrete mixture proportions were selected. The control contained 100% cement, the three binary mixes contained only BA as a partial substitute of 10, 20, and 30% cement (BA10, BA20, BA30), and the remaining three ternary mixes contained BA and SF. In ternary mixes, a large amount of cement ranging from 30% (25% BA + 5% SF) to 40% (33% BA + 7% SF) and 50% (40% BA + 10% SF) was substituted. Complete details of the ingredients of each mixture and details of the test specimens are given in Table 3.

**Table 3:** Mixture proportions of concrete samples for compressive and tensile tests, water absorption, and porosity (w/b = 0.35; a/b = 3.37; s/a = 0.40).

Mix ID (Total # of specimens for each = 18 and cured for 7, 28, 91 days)	W (kg/m^3^)	Binder, b (kg/m^3^)			Aggregates, a (kg/m^3^)			Superplasticizer (% of b)	Slump (mm)
		C	BA	SF	FA (S)	CA 10 mm	CA 20 mm		
Control concrete (CC)	160	457	–	–	617	462	462	1.0	120 ± 30
Concrete containing 10% BA (BA10)		411	46	–				1.0	
Concrete containing 20% BA (BA20)		366	91	–				1.0	
Concrete containing 30% BA (BA30)		320	137	–				1.1	
Concrete containing 25% BA and 5% SF (BA25SF5)		320	114	23				1.2	
Concrete containing 33% BA and 7% SF (BA33SF7)		274	151	32				1.3	
Concrete containing 40% BA and 10% SF (BA40SF10)		229	182	46				1.4	

The primary objective of adding SF to ternary mixtures was to maximize the percent cement replaced with BA without compromising other mechanical and durability-related properties. Therefore, to ameliorate the strength of binary concrete, compared to control concrete (CC), SF is added at 5 to 10% to mixes containing a high percentage of BA (25% or more). A constant water-to-binder (w/b) ratio of 0.35 was maintained for all the mixes. Since the total binder content for each mix was 457 kg/m^3^, and therefore according to w/b ratio the water content of all mixes was kept same to 160 kg/m^3^. However, the targeted workability corresponding to slump values of 120 ± 30 mm was achieved by additionally employing various percentages (wt.% of binder) of a naphthalene-based high-range water-reducing admixture (Table 3).

### Methods

#### Concrete mixing and preparation of specimens

A power-driven rotating pan mixer was used to mix the concrete ingredients, in accordance with the guidelines given by ASTM C192. Immediately after mixing and testing to achieve the required slump, cylindrical concrete specimens 100 mm in diameter and 200 mm in height were cast according to the standard method specified by ASTM C39. A total of eighteen specimens were cast for each mix to test three identical specimens with respect to aging (7, 28, and 91 days) to determine the compressive and splitting tensile strengths. After fabrication, the cylindrical molds were covered by plastic sheets and stored at the standard laboratory temperature (20 ± 1 °C) and relative humidity (60 ± 5%) for 24 h. All the concrete specimens were demolded upon completion 24 h after casting and moist-cured in a curing tub until testing on 7, 28, and 91 days. Upon reaching the desired curing period, both the upper and the lower ends of the concrete specimens were smoothly levelled by employing an end surface grinder.

#### Compressive and splitting tensile strength tests

**Figure 4:**
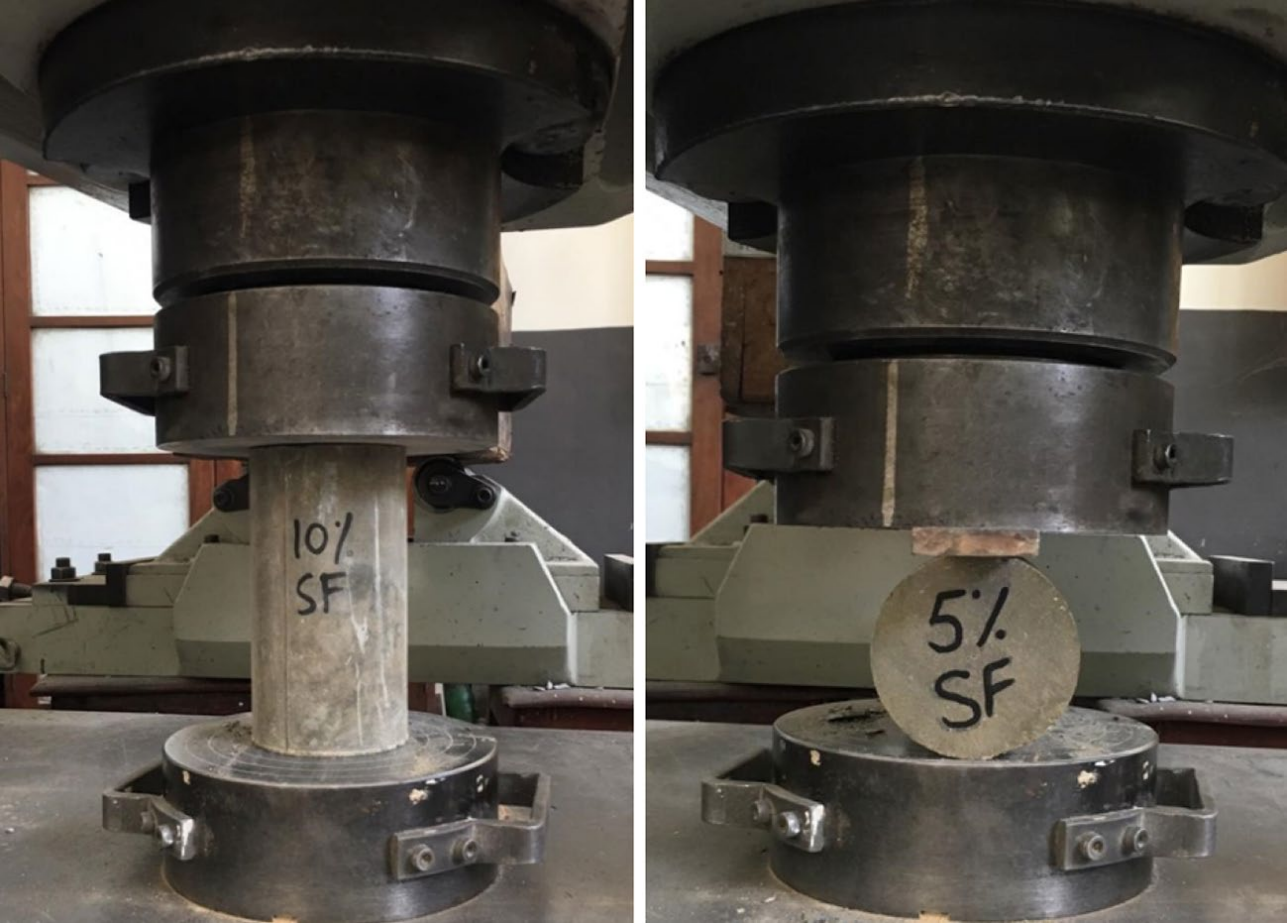
Test setup used to measure the compressive and tensile strength of concrete specimens.

The compression strength test was conducted on the cylindrical specimens as per ASTM C39 by deploying a 200-ton-capacity universal testing machine (UTM) assembly at a loading rate of 0.2 MPa/s. In contrast, the split tensile test was conducted as per ASTM C496 by deploying a 200-ton UTM at a loading rate of 1 MPa/min. Figure 4 shows the test setup used to measure the compressive and tensile strength of concrete specimens.

#### Water absorption

In addition to compression and tensile tests, WA tests were also performed on hardened concrete samples of all mixes according to ASTM C948. To perform WA tests, concrete cylindrical samples with a diameter of 100 mm and thickness of 50 mm were obtained by cutting 91-day moist-cured concrete specimens. The cut concrete samples were soaked in water at 21 °C and periodically weighed at a constant interval of 24 h until saturated-surface dry (SSD) weight stabilization was achieved. The SSD weight was deemed stabilize when less than a 0.5% difference in weight occurred between two consecutive readings. The latest yielded weight of the samples is referred to as “B”. Subsequently, the mass of the specimens suspended in water was measured to the nearest 0.01 g and referred to as "A". Note that the concrete samples were oven-dried at 100–110 °C and that weights were recorded every 24 h. After attaining a weight loss < 0.5% of the last measured weight, the sample was cooled inside a vacuum desiccator at room temperature. The sample weight recorded at room temperature is denoted “C”. Finally, the WA and AP can be calculated by using the following equations:

$$ {\text{Water}}\,{\text{Absorption }}\left( \% \right) \, = \, \left( {{\text{B }}{-}{\text{ C}}} \right)/{\text{C}} \times {1}00, $$

$$ {\text{Apparent}}\,{\text{Porosity }}\left( \% \right) \, = \, \left( {{\text{B }}{-}{\text{ C}}} \right)/\left( {{\text{B }}{-}{\text{ A}}} \right) \times {1}00. $$

#### Preparation of paste samples for microstructural testing

Paste specimens of the control mix (100% cement) and the mixes with different contents of BA (binary) and BA and SF (ternary), as partial replacements of cement (by weight), were prepared. For all mixes, a paste of standard consistency was obtained by mixing inside a Hobart mixer. Immediately after mixing, the fresh cement pastes were poured into small plastic containers (diameter of 20 mm and height of 50 mm) for casting. Subsequently, the plastic containers were capped and sealed prior to curing. After 91 days in the cured state, the specimens were dried to inhibit the process of hydration with the solvent exchange method. Finally, the flaky slices alongside the powder specimens were subjected to washing using isopropanol for 15 min. Afterwards, the paste samples were dried in an oven for 30 min at 40 °C to remove the isopropanol present in the samples, and the specimens were stored in sealed plastic bags prior to testing.

#### Scanning electron microscopy/energy-dispersive X-ray spectroscopy (SEM–EDS) analysis

For all the concrete mixes, SEM–EDS analysis was conducted on hardened fragments in the form of slices of the cement paste with a JSM-IT100 scanning electron microscope. Notably, the solvent exchange method was employed to dry the fragments of the hardened paste using isopropanol. Afterwards, the changes in the morphology and composition of all the specimens were studied.

#### Nitrogen adsorption isotherm technique

Similar to SEM–EDS, a N_2_ sorptiometry study was also conducted on the paste samples of all the studied concrete mixes. N_2_ sorptiometry was used to characterize the surface area and pore structure of the powdered specimens (weighing approximately 0.3 g) obtained from the 91-day cured hardened paste with a N_2_ sorption analyzer (NOVA2200e, Quanta chrome, USA) at 273 K. During the process, first, the specimens were degassed to remove absorbed contaminants from the open air. Consequently, N_2_ adsorption of the specimens was performed at ambient temperature and controlled pressure.

#### Fourier transform infrared (FTIR) analysis

The different phases of a variety of cement pastes were additionally characterized for all the studied mixes by performing FTIR using a Perkin Elmer Spectrum Two FTIR spectrometer. The powdered samples of all the studied mixes were dried and subjected to an infrared light source, and the infrared spectra were recorded in the wavenumber range of 400 to 4000 cm^−1^.

#### Thermogravimetric analysis (TGA) of cement pastes

TGA was also conducted on all the studied concrete mixes. The dried powdered specimens obtained from 91-day cured pastes were placed inside the ceramic vessel of a thermal gravimetric analyzer. To dry the specimens, they were heated inside the thermal gravimetric analyzer to 20 to 1000 °C at a rate of 10 °C/min, with N_2_ acting as the medium under static conditions. Additionally, alumina powder was incorporated as a reference to attain stability at elevated temperatures. Finally, the weight loss of various paste specimens in various temperature ranges was depicted by a comparison plot drawn using the built-in software.

## Results and discussion

### Influence of BA and SF on the evolution of the compressive and tensile strengths of concrete

Table 4 lists the compressive and splitting tensile strengths evolution results of all the tested concretes at 7, 28, and 91 days. These results are expressed at each age in terms of average of three identical specimens ± the corresponding standard deviation values.

**Table 4:** Compressive and tensile strengths of concrete with respect to aging.

Concrete specimen ID	Compressive strength, MPa^a^			Splitting tensile strength, MPa^a^		
	7 day	28 day	91 day	7 day	28 day	91 day
C	38.3 (± 2.22)	41.1 (± 1.72)	42.8 (± 1.71)	2.53 (± 0.14)	2.74 (± 0.06)	2.92 (± 0.17)
BA10	31.4 (± 0.52)	34.2 (± 2.24)	40.0 (± 0.24)	2.58 (± 0.13)	2.91 (± 0.11)	3.01 (± 0.19)
BA20	41.0 (± 0.38)	43.5 (± 2.03)	44.8 (± 1.14)	2.85 (± 0.18)	2.92 (± 0.21)	3.04 (± 0.19)
BA30	33.0 (± 3.37)	40.6 (± 1.77)	40.9 (± 1.78)	2.59 (± 0.17)	3.09 (± 0.13)	3.14 (± 0.12)
BA25SF5	33.8 (± 1.61)	37.3 (± 2.09)	40.3 (± 2.44)	2.74 (± 0.08)	2.82 (± 0.03)	3.01 (± 0.06)
BA33SF7	38.5 (± 1.01)	40.9 (± 0.99)	42.4 (± 0.40)	3.01 (± 0.14)	3.22 (± 0.06)	3.23 (± 0.11)
BA40SF10	22.5 (± 0.61)	35.6 (± 0.71)	37.2 (± 1.55)	2.17 (± 0.14)	2.80 (± 0.02)	2.88 (± 0.03)

^a^Values in parenthesis indicate the standard deviation of all test results.

**Figure 5:**
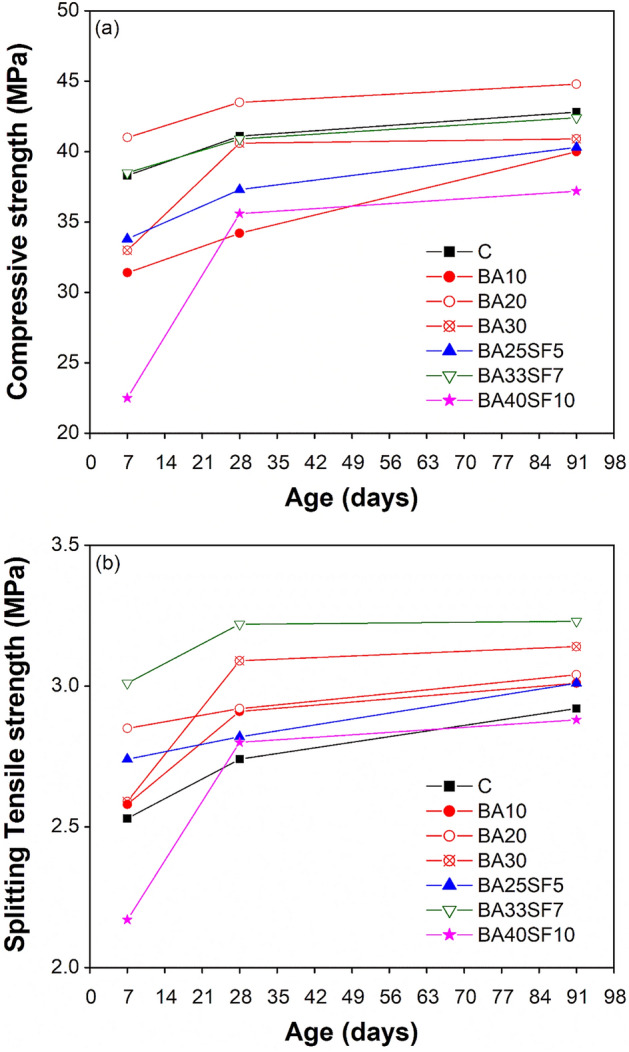
Comparison of strength evolution results between control and other concrete samples of binary and ternary mixes: (**a**) compressive strength, and (**b**) splitting tensile strength.

Figure 5a shows a comparison of the compressive strength results of CC and concrete where cement partially replaced with different percentages of BA and blends of BA and SF. In binary mixes with BA alone, up to 30% cement (10%, 20%, and 30%) was replaced, while in the ternary mixes with BA and SF, the cement substitution percentage was increased up to 50% (30%, 40%, and 50%) due to the highly reactive nature of SF. The purpose of studying ternary mixes of BA and SF was to compare their results to those of the control and binary mixes to determine the maximum replacement percentage of cement that does not compromise the strength. The concrete specimens were all water cured at a standard curing temperature of 20 °C until the age of testing.

The results show that the binary concrete containing 20% BA exhibited highest compressive strengths among all mixes, including those of the CC, irrespective of aging. However, the compressive strength of the other two binary mixes containing BA alone was lower than that of CC at all ages. However, the reduction was more significant for the binary mix containing a low percentage of BA (10% BA) than for the binary mix containing a higher percentage of BA (30% BA). The low compressive strength of the binary mix with only 10% BA is due to its lower degree of pozzolanic activity than mixes with high percentages of BA. Although a significant reduction in strength was observed for these binary mixes at early ages, an enhancement at later ages, especially at 91 days, was noticeable due to their late pozzolanic reactions. Moreover, the compressive strength of the binary mix containing 30% BA was comparable to that of CC.

To achieve high sustainability, efforts were made to regain the reduction in the compressive strength of binary mixes containing high percentages of BA by adding different percentages of SF. The test results show that no improvement in strength occurred in ternary mixes with an equal percentage of cement substitution (25% BA along with 5% SF) to that of the corresponding binary mix (30% BA). However, an encouraging response was noticed for ternary mixes containing high percentages of BA and SF (33% BA along with 7% SF), where the compressive strength was comparable to that of CC at all ages. The above results demonstrated fast early-age hydration and better packing and filling abilities due to the addition of very fine SF along with the later-age pozzolanic reaction of BA. With a further increase in cement substitution (50%), once again, a significant reduction in strength was observed in the ternary mix containing 40% BA and 10% SF. Note that among all the tested binary and ternary mixes, the lowest compressive strength results were observed for this ternary mix, which is attributed to its high percentage of cement substitution. This is because a high percentage of cement substitution affected both the early hydration and later-age pozzolanic reaction due to the production of less Ca(OH)_2_.

Unlike the compressive strength, all binary and ternary concrete mixes had higher splitting tensile strength than CC irrespective of aging (Fig. 5b), except at an early age (7 days) for the ternary mix with 50% cement substitution. Similar to the compressive strength, both the binary (BA20) and ternary (BA33SF7) mixes showed significantly higher splitting tensile strength than CC, irrespective of aging. Among all the mixes, the highest splitting tensile strength was demonstrated by the ternary mix with 40% cement substitution (BA33SF7).

In summary, the results demonstrated that the compressive strength of binary concrete mixes decreased at both low (10%) and high (30%) cement substitution percentages and that the best compressive strength results were achieved when 20% BA was used. Similar to that of binary mixes, the compressive strength of ternary mixes with low (25% BA + 5% SF) and high percentages of cement substitution (40% BA + 10% SF) decreased, and the best strength results were achieved when 33% BA was used along with 7% SF.

### Comparison of the water absorption and apparent porosity of control concrete and binary and ternary concrete mixtures

**Figure 6:**
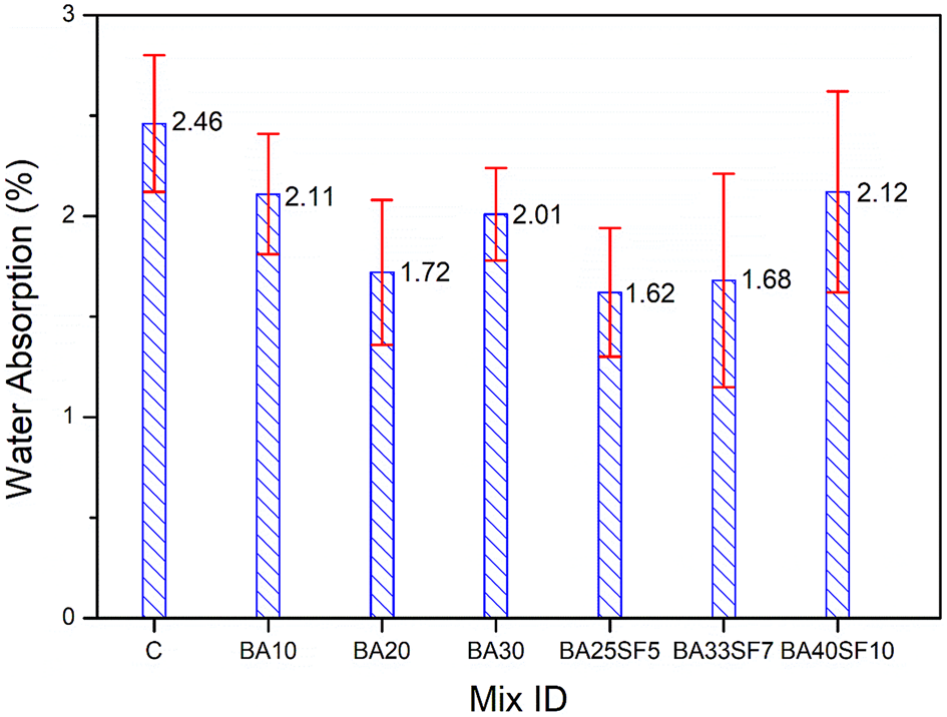
Comparison of water absorption results between control and other concrete samples of binary and ternary mixes after 91 days of standard curing.

**Figure 7:**
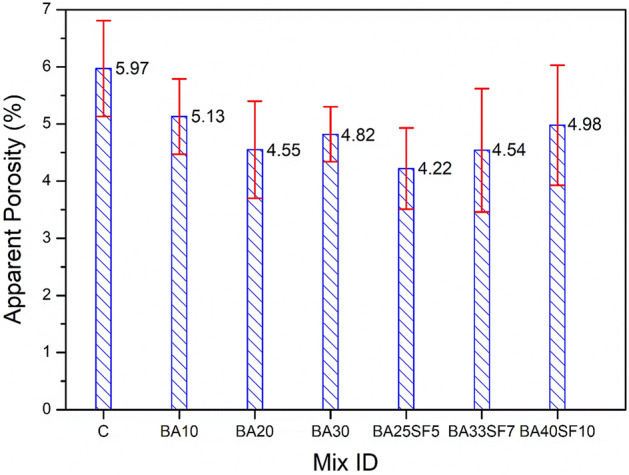
Comparison of apparent porosity results between control and other concrete samples of binary and ternary mixes after 91 days of standard curing.

In addition to the strength characteristics of the studied binary and ternary concrete mixtures, other important properties, such as WA and AP, were also measured and compared to those of CC. For the hardened concretes, WA and AP can be used as indirect indicators for the evaluation of their durability. Figures 6 and 7 show the effect of replacing different percentages of cement with BA alone (10, 20, and 30) or BA and SF (30, 40, and 50%) on WA and AP. Irrespective of the amount of cement replaced, all the studied binary and ternary concrete mixes exhibited lower WA than CC (Fig. 6). The lower WA of binary and ternary mixes compared to that of CC can be attributed to the binary and ternary mixes having lower AP values (Fig. 7). The current results indicated that the WA of binary mixes decreases with increasing percentage of BA up to a certain level of cement replacement (20%). For instance, a binary concrete mixture containing 30% BA demonstrated a slightly higher WA than that containing 20% BA (Fig. 6). A similar trend of AP values can also be observed in these binary concrete mixtures (Fig. 7). This can be attributed to the slower rate of the pozzolanic reaction of the concrete mixture with a high percent of cement replaced with BA. However, unlike binary concrete mixtures, a remarkable improvement in terms of lowered WA and AP values can be noted in ternary concrete mixtures with a high percent of cement replaced with BA and SF. The results indicated that the ternary mix with 30% (BA25SF5) cement replaced exhibited lower WA and AP than the corresponding mix (BA30) and other binary mixes (BA10 and BA20). However, a slight increase in WA and AP occurs in ternary mixes with increasing percent replacement of cement from 30 to 40 or 40 to 50%. However, the ternary mix with an even higher percent replacement of cement, such as 40% (BA33SF7), still exhibited lower WA and AP than the binary mixes with a relatively lower percent replacement of cement (10, 20 or 30%). This can be attributed to significant pore refinement in ternary concrete mixtures due to the very fine size of SF particles compared to BA. The slightly high values of WA and AP in a ternary mixture (BA40SF10) with a very high percent replacement of cement (50%) can be related to its slower rates of pore refinement and pozzolanic reaction.

### Prediction models to evaluate tensile strength based on compressive strength of concrete

Being a common engineering practice, values of experimental splitting tensile strength of concrete can be correlated with their corresponding compressive strength. This is because the general trend of both compressive and tensile strength developments remains consistent, irrespective of type of mixture proportions, binder type and content, aging, curing conditions and so on. In addition to existing codes such as ACI 318^57^, ACI 363^58^, CEB-FIP model code 1990^59^, numerous researchers have developed their own correlation between both properties according to different experimental data due to their specific type of concretes, geometry of specimen, curing conditions, and testing conditions^60–66^. However, among all, including existing codes, the equation used for this correlation remained consistent as [${f}_{sp }=a \left({f}_{c}^{^{\prime}}\right){ }^{b}$]. In this equation, ${f}_{c}^{^{\prime}}$ is known compressive strength from experimental results in MPa, while ${f}_{sp}$ represents the unknown splitting tensile strength of concrete to be predicted in MPa. The other two parameters (*a* and *b*) are the constants, where *b* grasp the dissimilarity of increasing rate among both properties as its value varies from one researcher to another according to their different test results. For instant, value of *b* is taken as 0.5, 0.67, and 0.71 by ACI 318^57^, CEB-FIP model code^59^, and Kim et al.^63^, respectively. The reason of different *b* values is because ACI 318 used specified compressive strength, CEB-FIP model code used compressive strength associated with the specific characteristic compressive strength, and Kim et al. used mean compressive strength of concrete. Moreover, the most existing correlations are developed using the 28 days experimental results and for conventional concrete subjected to standard curing (moist-cured under 20 °C) using Type-I cement. However, it is worth mentioning that, other than the characteristic or mean compressive strength of concrete, the variation of the exponent *b* can also be influenced by several other factors such as the type of cement, water to binder ratio, curing temperature, aging and so on^67^. Although some researchers have considered the effect of different influencing factors on the rate of both properties such as Kim et al.^63^ had proposed their correlation according to different binder types, curing, and aging. However, the effects of other influencing factors such as seasonal variations, geometry of specimen, type of concrete using SCMs was not considered. Therefore, it is necessary to evaluate the different existing correlations to determine the splitting tensile strength of different concretes produced in this study with different percentage of BA alone (BA10, BA20, BA30) and BA with SF (BA25SF5, BA33SF7, BA40SF10).

**Figure 8:**
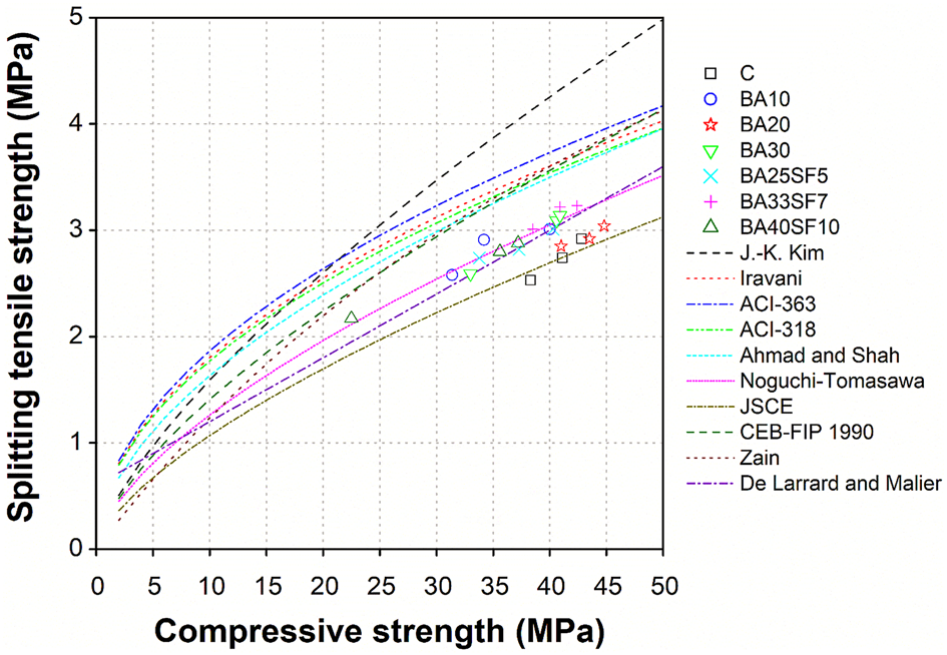
Comparison of experimental tensile and compressive strength relationships of different concrete mixtures with existing prediction models.

As shown in Fig. 8, a comparison of experimental compressive and splitting tensile strength relationship was drawn for various concrete specimens (control, binary and ternary mixes) with aging (3, 7, and 28 days), and compared to existing models used to evaluate tensile strength based on compressive strength^57–66^. Table 5 shows the general equation and different values of *a* and *b* corresponding to each existing model code. For all the prediction models, the values of splitting tensile strength, presented in Fig. 8, were estimated using the experimental results of compressive strength. The comparison of experimental and prediction results showed that all models presented on Fig. 8 overestimates splitting tensile strength significantly, except those proposed by JSCE-2012 design codes^65^, Noguchi-Tomosawa^64^, and De Larrard and Malier^66^. It can be clearly seen in Fig. 8 that these models best fit within the area of obtained experimental results. More specifically, based on the comparison of experimental compressive-tensile strength relation with those of prediction models, it seems that the JSCE-2012 prediction model [${f}_{sp }=0.23 \left({f}_{c}^{^{\prime}}\right){ }^{2/3}$] closely estimates the splitting tensile strength for control and a binary mix containing 20% BA (BA20) for any known value of compressive strength, irrespective of aging. Whereas, the Noguchi-Tomosawa model [${f}_{sp }=0.291 \left({f}_{c}^{^{\prime}}\right){ }^{0.637}$] closely estimates for all the remaining binary (BA10, BA30) and ternary mixes containing BA with SF (BA25SF5, BA33SF7, BA40SF10). In addition to Noguchi-Tomosawa model, a model proposed by De Larrard and Malier [${f}_{sp }=0.6+0.06 \left({f}_{c}^{^{\prime}}\right)$] also properly estimates the splitting tensile strength for these mixes for any value of compressive strength.

**Table 5:** Model equation and values of *a* and *b* corresponding to existing model codes.

Model code	Model equation	Values of	
		a	b
ACI 318^57^	${f}_{sp }=a \left({f}_{c}^{^{\prime}}\right){ }^{b}$	0.56	0.5
ACI 363^58^		0.59	0.5
CEB-FIP 1990^59^		0.301	0.67
Iravani^61^		0.57	0.50
Ahmad and Shah^62^		0.46	0.55
Kim et al.^63^		0.31	0.71
Tomosawa et al.^64^		0.291	0.637
JSCE-2012^65^		0.23	0.667
Zain et al.^60^	${f}_{sp }={f}_{c}^{^{\prime}}/[a \left({f}_{c}^{^{\prime}}\right)+$ *b*]	0.10	7.11
Larrard and Malier^66^	${f}_{sp }={a+b (f}_{c}^{^{\prime}})$	0.60	0.06

**Figure 9:**
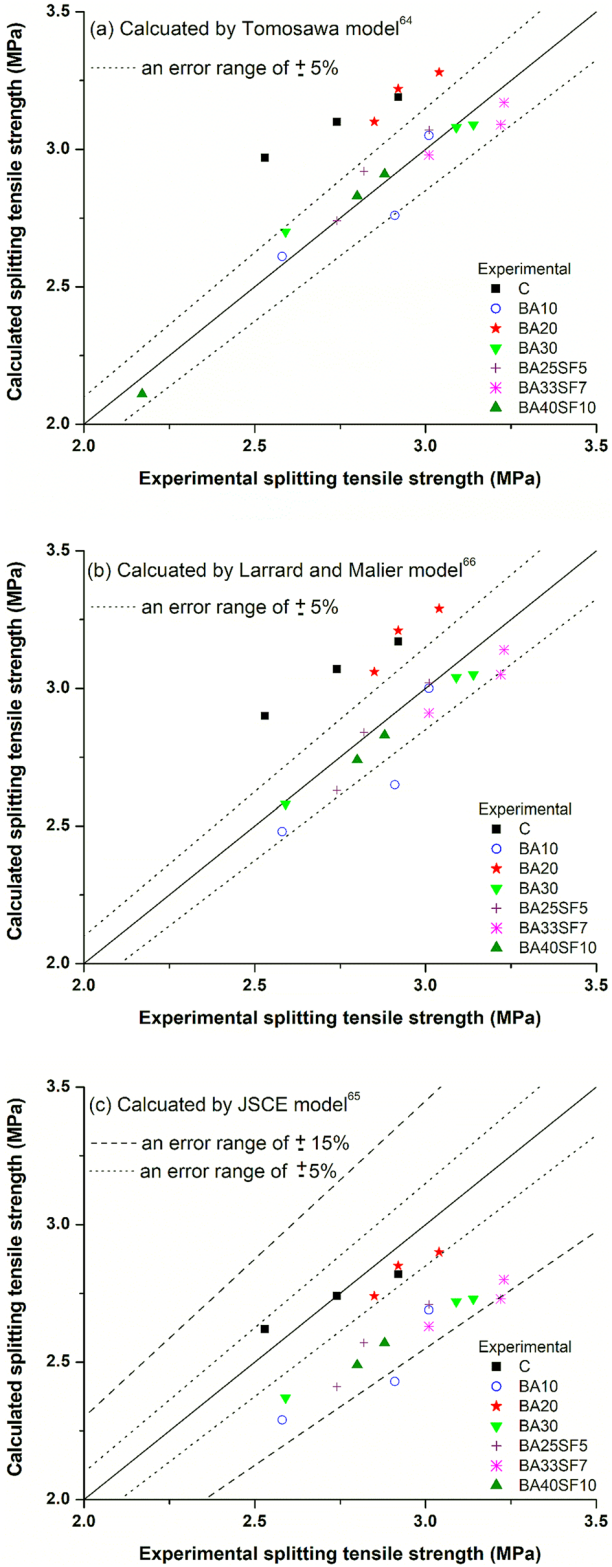
Comparison between experimental and predicted tensile strength results.

The above findings suggest that aging and the binder type do not significantly affect the correlation between splitting and compressive strength of concrete. These findings are in line with those noticed by Kim et al.^63^, where the relationship between splitting tensile strength and compressive strength of concrete was reported as independent of aging, cement type, and curing temperature. Consequently, it is proposed that the Noguchi-Tomosawa model^64^, or the model proposed by De Larrard and Malier^66^ be safely used to determine splitting tensile strength of all the tested concretes, within an error range of ± 5% (Fig. 9a,b), except for control and BA20 concretes. The JSCE model^65^ can be safely used to determine splitting tensile strength for control as well as BA20 concretes within an error range of ± 5% (Fig. 9c). Furthermore, Table 6 presents the summary of experimental results of splitting tensile strength as well as those calculated based on the prediction equations of closely estimating models of Noguchi-Tomosawa^64^, De Larrard and Malier^66^, and JSCE^65^. A ratio of experimental/predicted splitting tensile strengths data is also presented in Table 6. The minimum and maximum values of this ratio ranged between 0.85–1.05, 0.87–1.11, and 0.97–1.20 with an average value of 0.97, 1.0, and 1.10 for Tomosawa, Larrard and Malier, and JSCE model, respectively. The above ranges of ratio and average values of ratio as unity for Larrard and Malier model (1.0), and close to unity for Tomosawa model (0.97) having an average correction factor of 3%, indicate their high prediction accuracy. On the other hand, a relatively low degree of accuracy observed for JSCE model due to a higher average value of ratio (1.10) having an average correction factor of 10%. Though conservative, the JSCE model be safely used to predict splitting tensile strength of all the tested concretes, however, with slight underestimated values within an error range of ± 15% (Fig. 9c). Since the splitting tensile strength is used as the criterion of crack control, the underestimation of the splitting tensile strength using JSCE model remains safe and conservative with respect to current experimental values.

**Table 6:** Comparison of experimental and predicted tensile strengths of concrete.

Concrete specimen ID	Age (days)	Experimental (MPa)		Predicted, $${f}_{sp, p} $$ (MPa)			Experimental/predicted ratio $${(f}_{sp }/{f}_{sp, p})$$		
		${f}_{c}^{^{\prime}}$	${f}_{sp}$	Tomosawa^64^	Larrard and Malier^66^	JSCE^65^	Tomosawa^64^	Larrard and Malier^66^	JSCE^65^
C	7	38.3	2.53	2.97	2.90	2.62	0.85	0.87	0.97
	28	41.1	2.74	3.10	3.07	2.74	0.88	0.89	1.00
	91	42.8	2.92	3.19	3.17	2.82	0.92	0.92	1.04
BA10	7	31.4	2.58	2.61	2.48	2.29	0.99	1.04	1.13
	28	34.2	2.91	2.76	2.65	2.43	1.05	1.10	1.20
	91	40.0	3.01	3.05	3.00	2.69	0.99	1.00	1.12
BA20	7	41.0	2.85	3.10	3.06	2.74	0.92	0.93	1.04
	28	43.5	2.92	3.22	3.21	2.85	0.91	0.91	1.03
	91	44.8	3.04	3.28	3.29	2.90	0.93	0.92	1.05
BA30	7	33.0	2.59	2.70	2.58	2.37	0.96	1.00	1.09
	28	40.6	3.09	3.08	3.04	2.72	1.00	1.02	1.14
	91	40.9	3.14	3.09	3.05	2.73	1.01	1.03	1.15
BA25SF5	7	33.8	2.74	2.74	2.63	2.41	1.00	1.04	1.14
	28	37.3	2.82	2.92	2.84	2.57	0.97	0.99	1.10
	91	40.3	3.01	3.07	3.02	2.71	0.98	1.00	1.11
BA33SF7	7	38.5	3.01	2.98	2.91	2.63	1.01	1.03	1.15
	28	40.9	3.22	3.09	3.05	2.73	1.04	1.05	1.18
	91	42.4	3.23	3.17	3.14	2.80	1.02	1.03	1.15
BA40SF10	7	22.5	2.17	2.11	1.95	1.83	1.03	1.11	1.18
	28	35.6	2.80	2.83	2.74	2.49	0.99	1.02	1.12
	91	37.2	2.88	2.91	2.83	2.57	0.99	1.02	1.12
Average							0.97	1.0	1.10

### SEM–EDS analysis of control (C), binary (C/BA) and ternary (C/BA/SF) cementitious pastes

**Figure 10:**
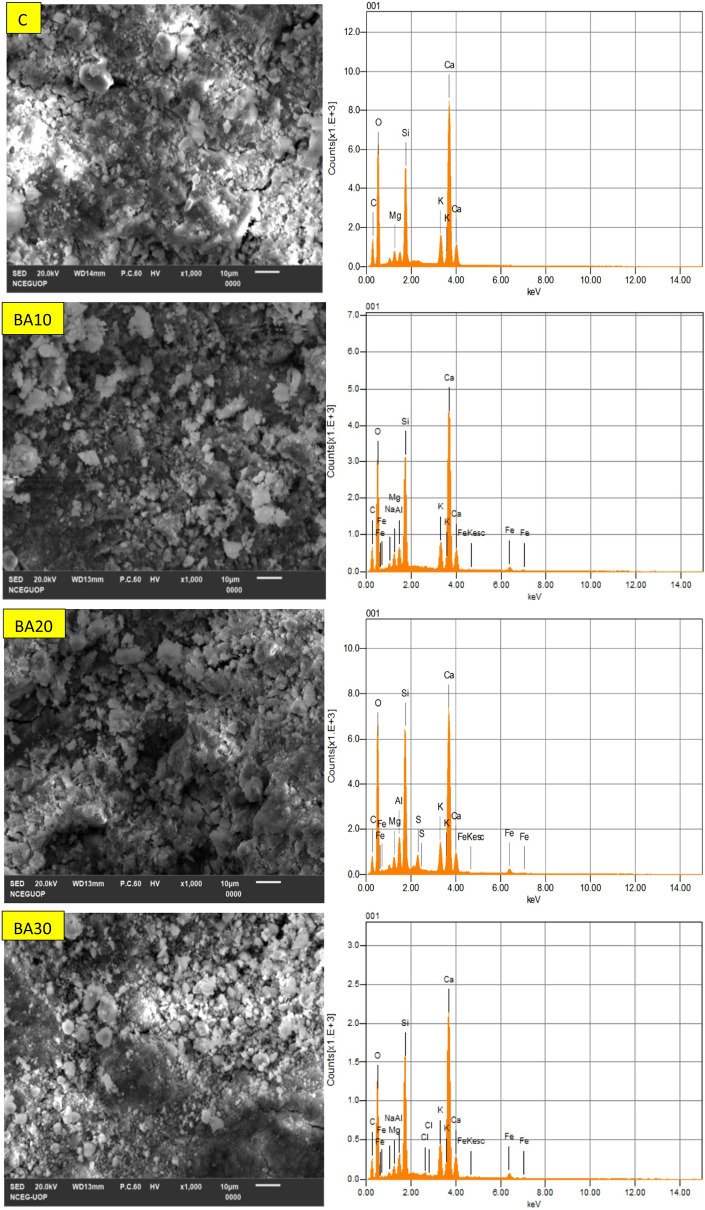

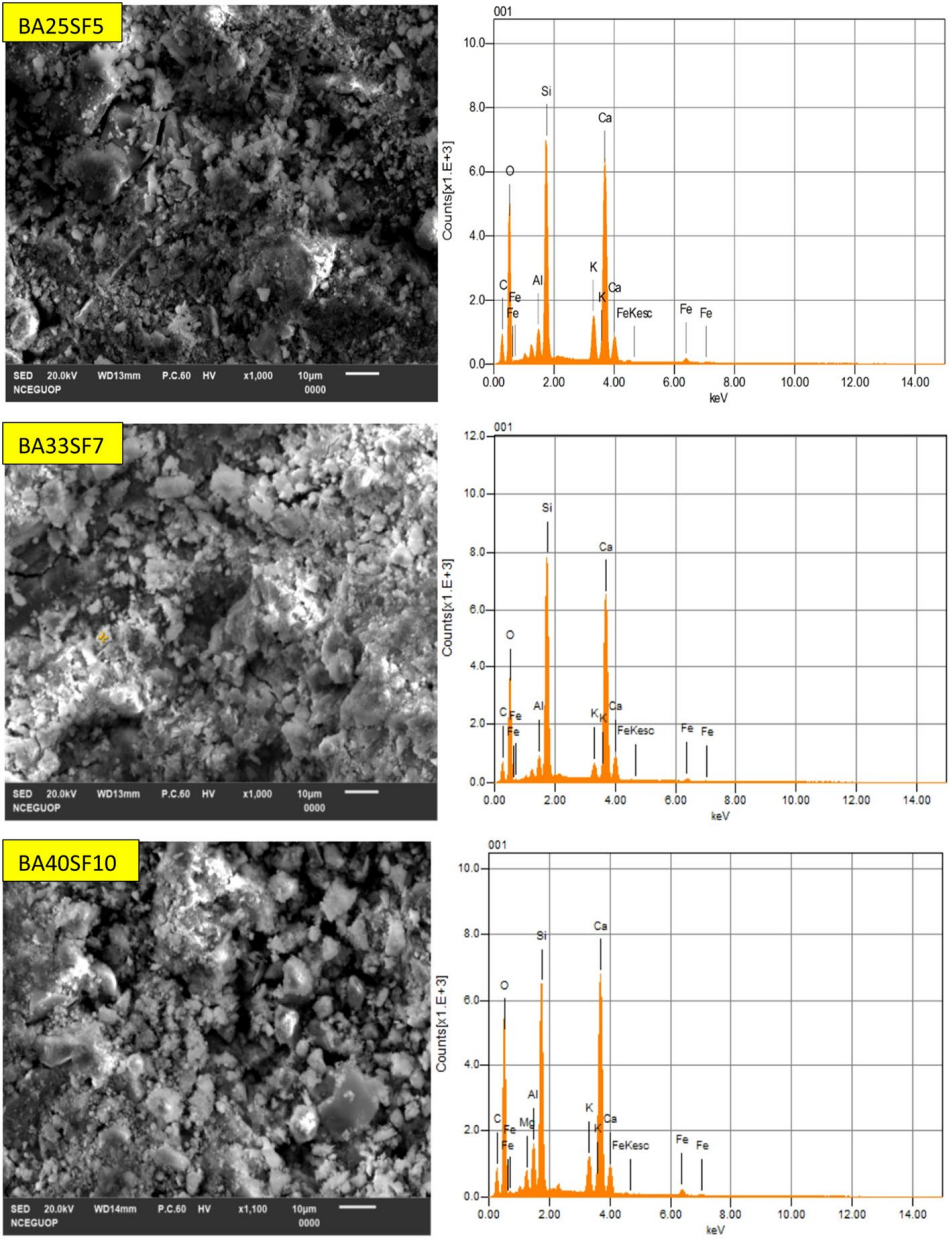
SEM–EDS spectrum of control and paste samples of binary (OPC-BA) and ternary mixes (OPC-BA-SF).

The microstructure of all the mixes used in this study was analyzed by performing SEM–EDS analysis on paste samples, as shown in Fig. 10. The EDS analyses were performed on SEM micrographs at different magnification levels for all mixes overall and at specific points to study the effect of BA and SF on the development and enhancement of the microstructure of cementitious pastes. The Ca/Si ratio calculated from EDS analysis for all the mixes is given in Table 7. Previous studies performed on cementitious mixes found that the Ca/Si ratio for the C–S–H phase ranges from 0.5 to 2, while a Ca/Si ratio greater than 2 represents C–H phases present in the paste sample^68–70^.

**Table 7:** Ca/Si atomic ratio from EDS analysis of control and mixes containing different percentages of BA alone and BA with SF.

Mix ID	Ca/Si atomic ratio		
	Overall	C–S–H phase	C–H phase
CC	1.93	1.73	3.30
BA10	1.74	1.54	3.06
BA20	1.35	1.34	2.73
BA30	1.68	1.46	2.93
BA25SF5	1.06	1.03	2.54
BA33SF7	1.00	0.91	2.41
BA40SF10	1.23	1.18	2.65

The SEM–EDS results show that the control mix has a higher Ca/Si ratio than all the other mixes containing BA or BA and SF. This may be attributed to the better pozzolanic properties and high fineness of both BA and SF, which causes densification of the paste matrix due to the formation of high-density C–S–H and C–H phases. Such results show the development of the microstructure due to incorporation of BA and its blend with SF in the cementitious matrix. The Ca/Si ratio of mixes with different amounts of BA shows that BA20 has the lowest Ca/Si ratio compared to BA10 and BA30. The results demonstrate that the optimum amount of BA used as a substitute for cement is approximately 20% for the development of stronger and more durable concrete. Other mechanical and microstructural testing results also revealed better performance by mixing 20% BA compared to the other two binary mixes containing only BA. Furthermore, the mixes with blends of both BA and SF (BA25SF5, BA33SF7, and BA40SF10) have the lowest Ca/Si ratios compared to all other mixes. Moreover, the blended mix with 40% cement replacement (BA33SF7) showed enhanced microstructure properties compared to the other two mixes with 30% (BA25SF5) and 50% (BA40SF10) cement replacement. In a similar manner to current study, several research studies in past have also proved an improved performance of concrete due to the addition of SF within a range of 5–10%^71–73^. The decrease in the Ca/Si ratio is attributed to the high fineness and reactivity of SF, which causes the formation of high-density C–S–H and C–H phases^74,75^. The formation of conventional high-density C–S–H and C–H phases cause densification and refinement of the microstructure, which would ultimately yield better performance when used in practical engineering applications.

### Fourier transform infrared (FTIR) analysis results of cement pastes

**Figure 11:**
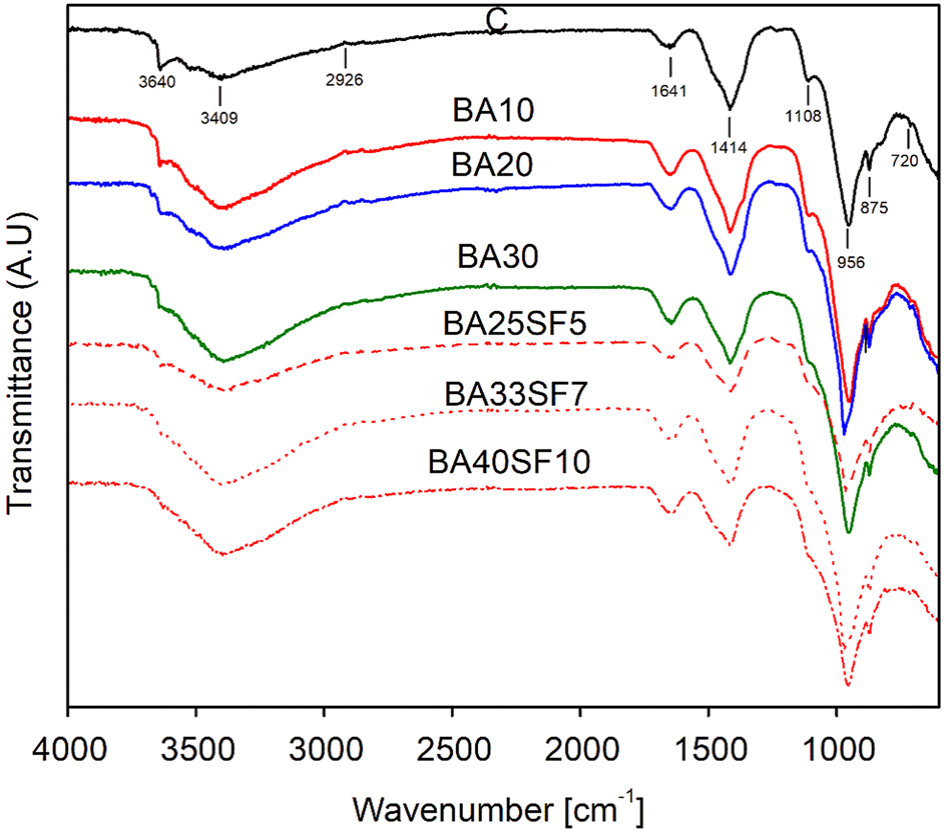
FTIR spectra of 91 days cured paste samples of control and other binary (OPC-BA) and ternary (OPC-BA-SF) concrete mixes.

Figure 11 compares the FTIR analysis results of the 91-day cured paste samples of the control and all the other binary and ternary mixes.

As illustrated in Fig. 11, the peaks between 900 and 1100 cm^−1^ are attributed to the vibrations of Si–O bonds in the C–S–H phase^76^. Compared to the control samples, the relative intensity of the (Si–O–Si) band is higher in paste samples with only BA or its blend with SF. The shift in the Si–O band toward a high wavenumber is due to the polymerization of silica. A slight shift (965 cm^−1^) is observed in the binary mix sample containing 20% BA. However, a significant shift (970 cm^−1^) is found in the ternary concrete samples with 5% SF (BA25SF5) and 7% SF (BA33SF7). The shift in the spectra of these binary (BA20) and ternary (BA25SF5 and BA33SF7) mixes indicated the formation of a large amount of high-density C–S–H gels. The formation of more C–S–H gels might be the possible reason for the development of the high compressive strength of these mixes. Additionally, the calcite formed due to carbonation are linked to the peaks at 720 cm^−1^, 875 cm^−1^, and 1415 cm^−1^.

The peak at 3645 cm^−1^ demonstrates the presence of free OH groups, which indicated the presence of the portlandite phase in all the tested paste samples^77^. This peak is more intense and visible in the control sample than in all other samples containing BA or SF. A relatively small peak is noticed for binary mix samples with different amounts of BA, which is reduced further for samples of ternary mixes with increasing percentages of SF. The peak remained very small or almost diminished in ternary mix samples with large amounts of SF (7% or 10%), which demonstrated their high pozzolanic reactivity due to the consumption of Ca(OH)_2_ in the paste samples^78^. The formation of gels of high-density C–S–H and the depletion of Ca(OH)_2_ due to the addition of BA and SF are also evident from the SEM–EDS and TGA results.

### Nitrogen adsorption results (surface area and pore structure of cement pastes)

**Figure 12:**
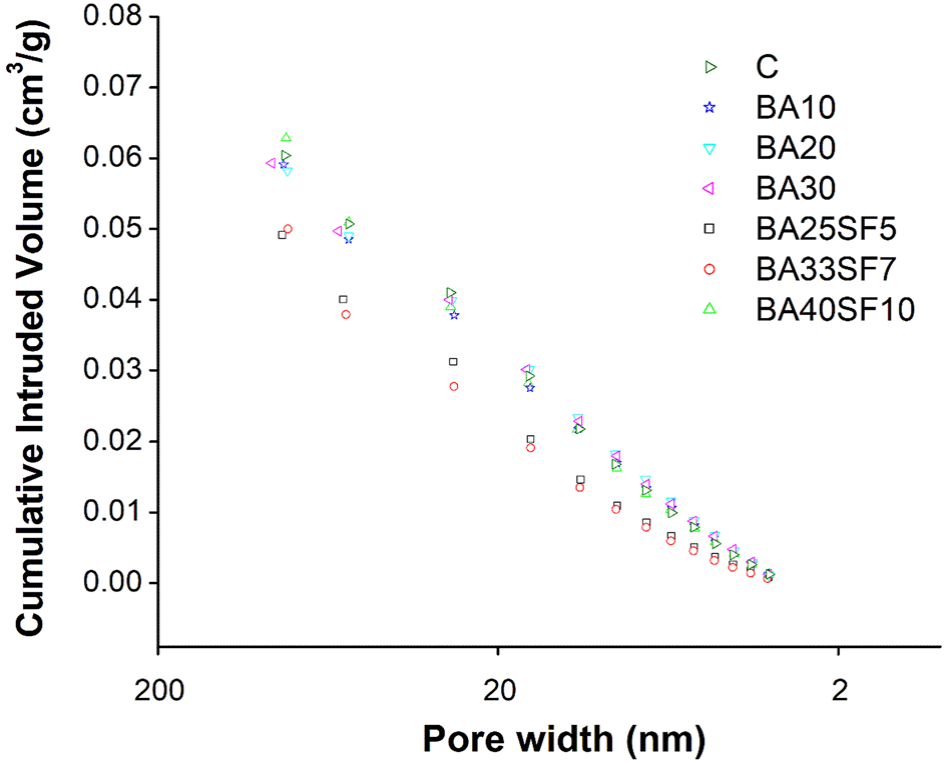
Comparison of the cumulative nitrogen intrusion volumes versus pore widths of all the tested paste samples (control, binary and ternary) subjected to 91 days of standard curing.

Figure 12 compares the cumulative nitrogen intrusion volumes versus pore widths of all the tested paste samples subjected to 91 days of standard curing. According to the test results, the paste of the ternary mix with 40% cement replacement (BA33SF7) exhibited the least amount of nitrogen volume intrusion (0.050 cm^3^/g), followed by the paste samples of the BA25SF5, BA20, BA30, BA10, Control and BA40SF10 mixes (Table 8). Despite the high percentage replacement of cement, it can be inferred that the lowest amount of nitrogen intruded in ternary mixes (BA33SF7 and BA25SF5) compared to the control or binary mixes, with the exception of the ternary mix with 40% BA and 10% SF. This is due to the densification of the pore structure, which can be attributed to the incorporation of highly reactive SF, which leads to improved pozzolanic reactivity and refinement of the pore structure^79,80^. Further, both BA20 and BA30 mixtures showed less nitrogen volume intrusion compared to the control samples, which could be explained by the densification of pore structures caused by the pozzolanic nature of BA. Among all the studied mixes, the maximum amount of nitrogen volume intruded in BA40SF10 is due to the presence of unreacted BA in the samples, which might have caused the increased porosity of the paste matrix^81,82^.

**Table 8:** Intruded pore volume for control and others samples having bagasse ash and bagasse ash with silica fume.

Mix ID	Intruded pore volume (cc/g)
CC	0.057
BA10	0.056
BA20	0.055
BA30	0.056
BA25SF5	0.052
BA33SF7	0.050
BA40SF10	0.058

**Figure 13:**
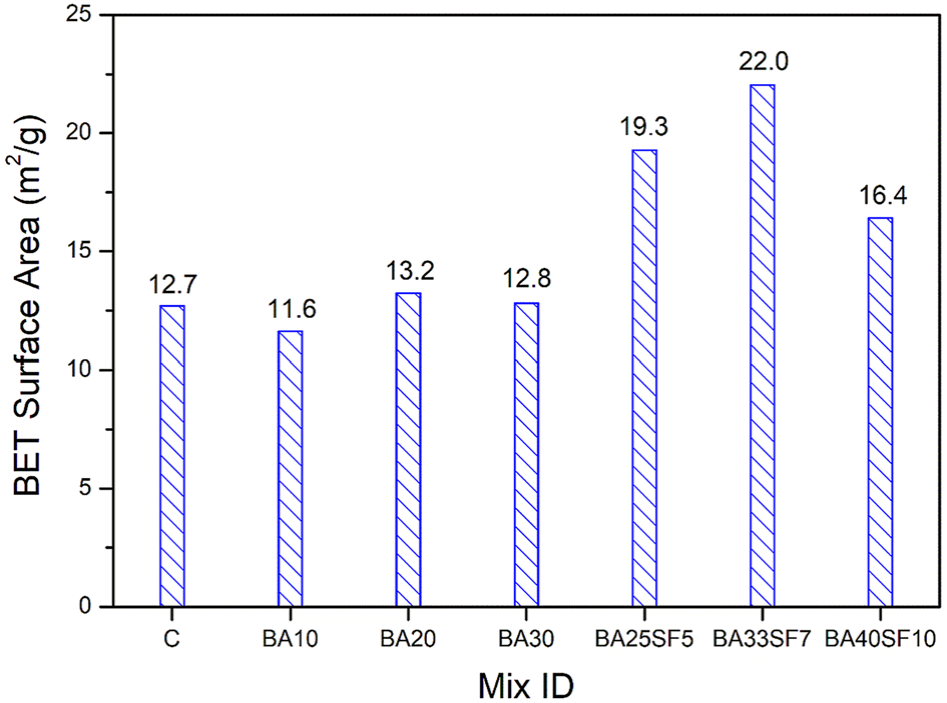
Comparison of BET surface area between control and other binary (OPC-BA) and ternary (OPC-BA-SF) concrete mixes.

Figure 13 compares the Brunauer–Emmett–Teller (BET) surface areas of control samples and the samples containing BA and blends of BA and SF. The ternary mix with 40% replacement of cement (BA33SF7) exhibited the largest surface area among all samples. According to past studies, an increase in surface area indicates an improvement in the microstructure characteristics of C–S–H gels^83,84^. In addition to the ternary mix, some binary mixes (BA20 or BA30) containing only BA also showed a slightly large surface area than the control sample. However, a decrease in surface area was observed in a binary mix when a very low percentage of BA (BA10) was incorporated. The above findings demonstrate substantial effects of different BA contents on the formation of pore structures.

A significant increase in the surface area of ternary mixes (BA25SF5 and BA33SF7) is attributed to the formation of highly dense C–S–H phases due to the presence of both BA and SF. This was demonstrated earlier through SEM–EDS analysis, which demonstrates a dense cementitious matrix with fewer pores. Among the ternary mixes, an exception also exists where the addition of a large volume of BA along with SF (BA40SF10) leads to a decrease in the surface area. The decrease in the surface area suggests the presence of unreacted BA in the paste matrix, which causes jamming of hydration products and ultimately leads to the formation of large pores and a more porous system^80^.

### Thermogravimetric analysis (TGA) results of cement pastes

**Figure 14:**
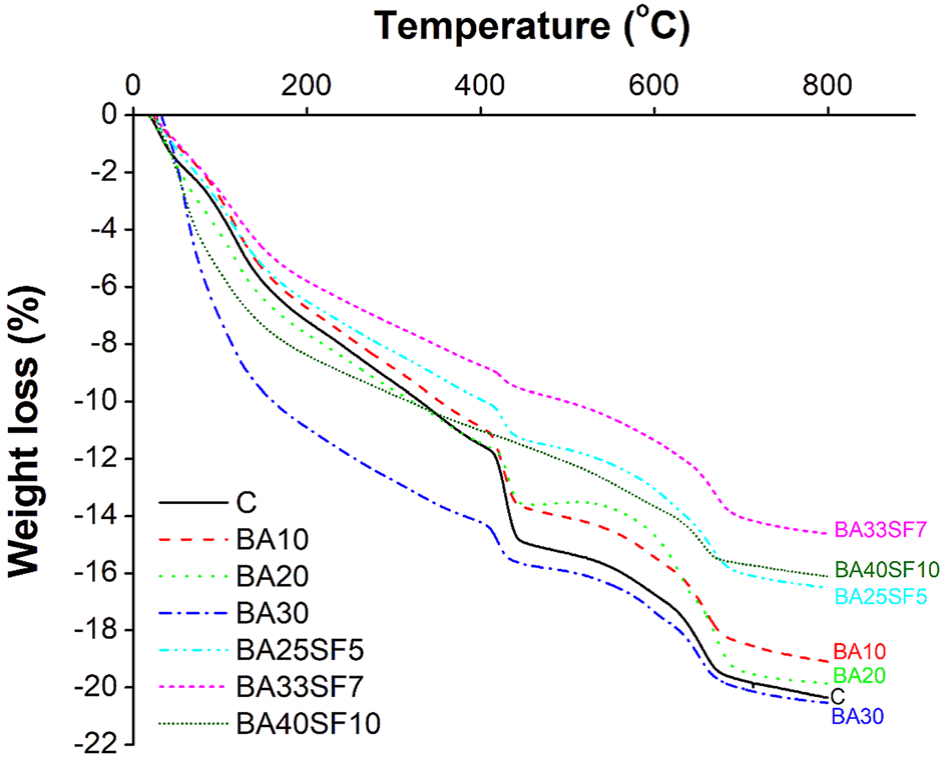
Comparison of thermogravimetric analysis of 91 days cured paste samples of control and other binary (OPC-BA) and ternary (OPC-BA-SF) concrete mixes.

Thermogravimetric analysis of cement paste samples was performed to evaluate the effect of BA and SF on the amount of Ca(OH)_2_, which was found from the weight loss between 400 and 500 °C^85^. Figure 14 shows a comparison of the thermogravimetric analysis results obtained for the 91-day cured samples of the control and other binary (BA10, BA20, and BA30) and ternary mixes (BA25SF5, BA33SF7, and BA40SF10).

Table 9 shows that the amount of Ca(OH)_2_ in the control sample (100% cement) is higher than that in all the other tested samples of binary and ternary mixes. The samples with different amounts of BA (10%, 20% and 30%), used as a cement substitute, show a decrease in the amount of Ca(OH)_2_, which is associated with the pozzolanic reactivity of BA reacting with Ca(OH)_2_ to form more C–S–H. The least Ca(OH)_2_ was recorded in ternary specimens with both BA and SF. This is attributed to the higher pozzolanic reactivity of amorphous silica present in SF, which leads to the formation of more C–S–H by utilizing the C–H phase, thus lowering the C–H content in the solution.

**Table 9:** Amount of calcium hydroxide (C–H) with respect to the content of binder and OPC after 91 days.

Mix ID	CH	CH/OPC
CC	18.5	18.5
BA10	15.2	16.9
BA20	9.80	12.3
BA30	8.40	12.0
BA25SF5	8.10	11.6
BA33SF7	5.80	9.70
BA40SF10	5.30	10.6

The normalized C–H content of all the binary and ternary mixes containing BA and SF was less than that of the control sample, as shown in Table 9. This is due to the increased number of nucleation sites and filling effect of mineral admixtures, as discussed in the FTIR and SEM–EDS analyses. In addition, the ternary mixes with both BA and SF show less normalized C–H than all the other samples, which is attributed to the high fineness and reactivity of SF. Interestingly, the ternary mix (BA40SF10) contains slightly more normalized C–H than BA33SF7. The results also indicate that the ternary mix (BA40SF10) consumes less C–H, which may be due to the incorporation of a large amount of BA in the mix; therefore, some part of the BA remains unhydrated.

## Conclusions

In this study, researchers have investigated the possibility of using a high-volume BA and its blend with SF as a partial replacement of cement to produce a sustainable and high-performance concrete. In addition to the control (100% cement), several binary mixtures prepared by partially substituting cement with BA (10%, 20%, and 30%) and ternary mixtures prepared by partially substituting cement with BA/SF (25%/5%, 33%/7%, and 40%/10%) were selected. The influence of high-volume cement replacement with BA alone and BA/SF on the hardened mechanical properties of concrete (compressive and tensile strengths, WA, and AP) was assessed and compared to that of the CC. In addition to concrete specimens, several paste samples for each mix were prepared to scientifically understand the influence of BA and SF on the matrix by performing micro and pore structure analyses using SEM–EDS, FTIR, TGA, and N_2_ adsorption techniques.

Following is a summary of major findings of this study:

The current results demonstrated that the compressive strength of binary concrete mixes decreases at both low (10%) and high (30%) cement substitution percentages and that the best compressive strength results were achieved when 20% BA was used. Similar to that of binary mixes, the compressive strength of ternary mixes with low (25% BA + 5% SF) and high percentages of cement substitution (40% BA + 10% SF) decreased, and the best strength results were achieved when 33% BA was used along with 7% SF. The higher strengths of these binary (20% BA) and ternary (33% BA + 7% SF) mixes are also validated by their lower WA and AP compared to other mixes.

A close agreement between experimental correlation of tensile and compressive strength to those of Noguchi–Tomosawa and the model proposed by De Larrard and Malier suggests that these models be used to reasonably estimate the tensile strength of all concrete mixes containing BA or BA with SF, except the control and the one containing 20% BA where use of JSCE model is recommended. Furthermore, the JSCE model can also be safely used to predict the tensile strength of all the concretes with their slightly underestimated values.

FTIR analysis confirmed the densification of the microstructure with the addition of BA alone or BA and SF. A prominent shift in peaks from 955 to 970 cm^−1^ was observed in the spectra of BA20 and BA33SF7, which indicates the formation of a large amount of C–S–H phases with a low Ca/Si ratio. The intensities of Ca(OH)_2_ peaks (3641–3644 cm^−1^) remarkably decreased with the incorporation of both BA and SF due to their high pozzolanic reactivity, which ultimately led to the formation of more C–S–H phases.

N_2_ adsorption analysis also showed that the pore structure densifies with the addition of BA alone or BA and SF to the cement paste matrix. A decrease in the intruded pore volume and an increase in the BET surface area of the paste matrix indicates an improvement in the pore structures of BA20, BA25SF5 and BA33SF7. However, an increase in the pore volume and a decrease in the surface area were observed for the high-volume replacement mixes (BA30 and BA40SF10). This might be due to unreacted BA, which ultimately causes high vascularity of the paste matrix.

The formation of high-density C–S–H and C–H phases was also observed in SEM–EDS analysis with the addition of BA alone or BA and SF, which caused densification of the microstructure. The Ca/Si ratios only slightly decreased with the addition of BA alone, while a significant decrease in Ca/Si ratios was found with the combined addition of BA and SF. Thus, the considerable decrease in the Ca/Si ratio with the incorporation of SF leads to the formation of high-density C–S–H and C–H phases, which causes densification and compaction of the microstructure and ultimately leads to better engineering performance.

TGA reveals a reduction in the amount of portlandite phase in the mixes with BA alone or BA and SF compared to that in the control sample. The decrease in the amount of portlandite phases is an indication of the high pozzolanic reactivity of both BA and SF, which utilizes the C–H phase to form more C–S–H phases and leads to the formation of more densified micro and pore structures.
